# Thyroid function in children with attention-deficit/hyperactivity disorder and specific learning disorder: a retrospective comparative study

**DOI:** 10.55730/1300-0144.6349

**Published:** 2026-01-29

**Authors:** Gül TRABZON, Seda Aybüke SARI, Servet YÜCE, Şeyma Demiray GÜLLÜ

**Affiliations:** 1 Division of Pediatric Endocrinology, Department of Pediatrics, Faculty of Medicine, Hatay Mustafa Kemal University, Hatay, Turkiye; 2 Department of Child and Adolescent Psychiatry, Faculty of Medicine, Hatay Mustafa Kemal University, Hatay, Turkiye; 3 Department of Public Health, İstanbul Arnavutköy District Health Directorate, İstanbul, Turkiye; 4 Department of Pediatrics, Faculty of Medicine, Hatay Mustafa Kemal University, Hatay, Turkiye

**Keywords:** ADHD, thyroid function, specific learning disorder, T_3_, T_4_, neuroendocrinology

## Abstract

**Background/aim:**

This study analyzes thyroid function parameters (free T_3_, free T_4_, TSH) in children with attention-deficit/hyperactivity disorder (ADHD), ADHD with specific learning disorder, and healthy controls, to determine whether subtle thyroid dysregulation contributes to the phenotypic heterogeneity of ADHD.

**Materials and methods:**

This retrospective, cross-sectional study included 135 children aged 6–18 years: 62 diagnosed with ADHD (38 isolated ADHD and 24 ADHD+SLD) and 73 healthy controls. Demographic, anthropometric, and thyroid function data (free T_3_, free T_4_, TSH) were extracted from medical records. Group comparisons were conducted using independent samples t-tests, ANOVA, and ANCOVA adjusted for age, sex, and treatment status.

**Results:**

Children with ADHD exhibited higher free T_3_ (p = 0.003), lower free T_4_ (p = 0.020), and higher TSH (p = 0.048) levels than the controls. Subclinical hypothyroidism, isolated T_3_ elevation, and euthyroid Hashimoto thyroiditis were more prevalent in the ADHD group (p = 0.032). No significant thyroid parameter differences were observed between isolated ADHD and ADHD+SLD. The direction of these endocrine differences persisted after covariate adjustment, although only free T3 remained statistically significant, suggesting subtle hypothalamic-pituitary-thyroid axis dysregulation in ADHD independent of stimulant treatment.

**Conclusion:**

Children with ADHD exhibit subtle alterations in thyroid function, characterized by higher T_3_ and TSH and lower T_4_ levels, which may reflect compensatory or metabolic imbalance rather than overt thyroid disease. These findings highlight the potential role of neuroendocrine modulation in the pathophysiology of ADHD.

## 1. Introduction

Attention-deficit/hyperactivity disorder (ADHD) is one of the most common neurodevelopmental disorders in childhood, characterized by pervasive symptoms of inattention, hyperactivity, and impulsivity that interfere with daily functioning and academic achievement.^1^ Beyond its behavioral manifestations, ADHD frequently cooccurs with other developmental conditions, among which specific learning disorder (SLD) is particularly prevalent[1]. The coexistence of ADHD and SLD not only compounds educational difficulties but also amplifies psychosocial challenges, underscoring the need for integrated multidisciplinary evaluation [2,3].

Although the etiology of both ADHD and SLD is multifactorial, encompassing genetic, neurobiological, and environmental influences, there is growing evidence that endocrine and metabolic pathways may also play a contributory role [4,5]. Thyroid hormones, in particular, are essential regulators of brain development, modulating neuronal differentiation, synaptogenesis, and cortical maturation [6]. Even mild alterations in thyroid function can disrupt cognitive domains such as attention, memory, and executive function—processes that are critically impaired in both ADHD and SLD [7].

Previous studies have reported associations between overt thyroid dysfunction, especially hypothyroidism, and impaired cognitive outcomes. However, data on the subtler spectrum of thyroid function test (TFT) abnormalities—including subclinical hypothyroidism, isolated T_3_ elevations, or autoimmune thyroiditis—in children with ADHD or SLD remain limited and inconclusive [7]. Some reports suggest that subclinical thyroid dysfunction may exacerbate attentional difficulties or learning impairments, while others fail to demonstrate a consistent relationship [8].

Given these uncertainties, exploring the relationship between thyroid function and neurodevelopmental conditions may yield clinically relevant insights. This retrospective study was designed to compare thyroid function parameters across three pediatric groups: children with ADHD alone, those with comorbid ADHD and SLD, and age- and sex-matched healthy controls. By delineating the endocrine profiles in these populations, we aim to clarify whether subtle thyroid dysfunction contributes to the heterogeneity of cognitive and behavioral phenotypes in affected children.

## 2. Materials and methods

### 2.1. Study design and participants

Included in this retrospective, cross-sectional study were children aged 6–18 years who were evaluated in child and adolescent psychiatry, pediatrics, and pediatric endocrinology outpatient clinics between January 2022 and January 2025. A total of 142 participants were initially screened; however, seven patients with isolated learning difficulties were excluded due to the small sample size, resulting in a final sample of 62 children diagnosed with attention-deficit/hyperactivity disorder (ADHD) and 73 healthy controls. Within the ADHD sample, two diagnostic subgroups were identified: isolated ADHD (n = 38) and ADHD with comorbid specific learning disorder (ADHD+SLD; n = 24). All psychiatric diagnoses were made by board-certified child and adolescent psychiatrists according to the Diagnostic and Statistical Manual of Mental Disorders (5th edition) criteria.

Children referred to the child and adolescent psychiatry clinic for various complaints and subsequently diagnosed with SLD and/or ADHD constituted the study group. Due to the retrospective design of the study, the healthy controls were not systematically screened using standardized psychiatric instruments but were rather selected from children who had never been referred to the child and adolescent psychiatry clinic and who had no documented psychiatric complaints in their medical records. The control group thus consisted of children who attended routine well-child visits in the pediatrics clinic and had no known medical or psychiatric conditions, as well as those referred to the pediatric endocrinology clinic for nonspecific concerns (e.g., screening for possible thyroid abnormalities) but who were confirmed after evaluation to have normal physical examination findings, normal thyroid function tests, and no chronic or acute medical comorbidities. The healthy control group, therefore, comprised children with no known chronic medical conditions, no regular medication use, and normal physical examination findings, and whose thyroid function test results obtained during routine clinical evaluations were all within the laboratory-specific reference ranges. Only children with thyroid function tests obtained during clinically stable periods were included, and those with acute infection, febrile illness, or any systemic inflammatory condition at the time of blood sampling were excluded from the study.

The study was conducted in a region known to be at risk for iodine deficiency, particularly in the aftermath of a recent earthquake when the public health infrastructure was disrupted. All study groups were recruited from the same geographical area and were therefore exposed to similar environmental and nutritional conditions. Thus, while iodine status may have influenced overall thyroid hormone levels, it is unlikely to have biased the between-group comparisons. The individual iodine status of the participants was not included in this retrospective dataset, but should be considered in future prospective studies.

As the study was conducted retrospectively, drawing from existing medical records, the requirement for informed consent was waived with the approval of the institutional ethics committee.

### 2.2. Data collection

Demographic characteristics, perinatal history, anthropometric measurements, laboratory parameters, thyroid function and thyroid ultrasonography findings, comorbidities, medication use, and family medical history were retrieved from the electronic medical records. The pharmacological treatment in the ADHD group was primarily stimulant medication, most commonly methylphenidate. Anthropometric indices (height, weight, body mass index (BMI), and standard deviation scores (SDS)) were calculated using standardized national references. Laboratory-specific reference ranges were used for the interpretation of thyroid function tests: free T_3_: 3.0–4.7 pg/mL, free T_4_: 0.86–1.40 ng/dL, and TSH: 0.67–4.16 μIU/mL. Cases identified with a thyroid hormone resistance-like pattern were defined based on the presence of inappropriately normal or elevated TSH levels in the context of elevated free T_3_ and free T_4_ concentrations, in the absence of overt clinical hyperthyroidism. Genetic confirmation was not available due to the retrospective study design. Isolated T_3_ elevation was defined as free T_3_ levels above the upper limit of the laboratory-specific reference range (4.7 pg/mL), in the presence of normal free T_4_ and TSH concentrations. All thyroid function tests were performed in the same central laboratory using a chemiluminescent immunoassay (CLIA) method. In patients with abnormal thyroid function test results, the measurements were repeated as part of routine clinical practice, and only abnormalities observed in at least two separate assessments were considered for analysis.

Thyroid ultrasonography, when available, was reviewed for gland size, parenchymal echogenicity and heterogeneity, presence of nodules or pseudonodules, and vascularity.

### 2.3. Handling of missing data and outliers

Variables with incomplete records were subjected to an available-case analysis; no imputation procedures were applied. Extreme values were examined visually (scatterplots and distribution curves) and retained if clinically plausible. Sample sizes varied across the analyses due to missing data, as indicated in the corresponding tables.

### 2.4. Statistical analysis

Statistical analyses were performed using IBM SPSS Statistics version 30.0 (IBM Inc., Armonk, NY, USA). The normality of continuous variables was assessed using the Shapiro–Wilk test and visual inspections of histograms and Q–Q plots. Continuous variables were presented as mean ± standard deviation (SD), and categorical variables as frequencies and percentages. For comparisons between the isolated ADHD and ADHD+SLD groups, independent samples t-tests were used for continuous variables. For comparisons among isolated ADHD, ADHD+SLD, and control groups, one-way analysis of variance (ANOVA) was applied. The homogeneity of variances was evaluated using Levene’s test. For adjusted group comparisons, analysis of covariance (ANCOVA) was performed in which age, sex, and medication status were covariates. When overall group differences were detected in one-way ANOVA, pairwise comparisons were conducted using Tukey’s Honestly Significant Difference (HSD) post hoc test, which controls for type I errors in multiple comparisons. Effect sizes were calculated as Cohen’s d for pairwise tests and eta squared (η^2^) for the ANOVA/ANCOVA models to quantify the magnitude of between-group differences. Categorical variables were compared using Pearson’s chi-square test. Fisher’s exact test was used for variables with expected cell counts <5. Statistical significance was defined as p < 0.05 (two-tailed).

### 2.5. Ethical considerations

The study protocol was approved by the Institutional Ethics Committee of Hatay Mustafa Kemal University (Approval date/no: 20/11/2024, 21). As this was a retrospective study based on existing records, the requirement for informed consent was waived.

## 3. Results

A total of 135 children were included in the analysis, including 62 patients with ADHD and 73 healthy controls. The demographic and anthropometric characteristics of the groups are presented in Table 1.

Children with ADHD were comparable to healthy controls in terms of age (11.44 ± 2.74 vs 11.97 ± 3.52 years, p = 0.857). Similarly, the sex distribution was comparable between the groups (41.9% vs 34.2%, p = 0.462), and birth weight and gestational age also did not differ significantly between the groups (p = 0.839 and p = 0.501, respectively).

As expected, none of the participants in the control group were undergoing treatment, whereas half of the children with ADHD were undergoing pharmacological therapy (p < 0.001).

Mode of delivery (normal vaginal delivery vs cesarean section) did not differ significantly between the two groups (p = 0.414), while history of incubator admission in the neonatal period was substantially more common in the ADHD group (27.9% vs 9.4%, p = 0.008).

Regarding growth parameters, the ADHD group had significantly lower height (135.85 ± 13.86 vs 149.05 ± 17.69 cm, p < 0.001) and weight (37.14 ± 14.99 vs. 44.84 ± 15.14 kg, p = 0.003). Although height SDS did not differ (p = 0.542), both weight SDS (p = 0.045) and BMI SDS (p = 0.022) were significantly lower in the ADHD group, while BMI values were comparable (p = 0.182).

In thyroid function tests, the ADHD group demonstrated lower free T_4_ levels (p = 0.020) and higher free T_3_ levels (p = 0.003) compared with the control group. TSH values were also significantly higher in the ADHD group (p = 0.048).

Thyroid ultrasonography data were available for 29 (46.8%) of the children with ADHD. Among those evaluated, ultrasonographic findings were normal in 10 patients (34.5%). Abnormal findings included increased thyroid vascularity in 14 patients (48.3%), thyroid nodules in four patients (13.8%), and parenchymal heterogeneity in one patient (3.4%). No cases of significant thyroid enlargement were documented.

Thyroid function classification differed significantly between groups (p = 0.032). Subclinical hypothyroidism, isolated T_3_ elevation, and euthyroid Hashimoto thyroiditis were more frequently observed among the children with ADHD, whereas the control group had predominantly normal thyroid function (Table 1).

### 3.1. Comparison of ADHD subgroups (isolated ADHD vs ADHD+SLD)

A total of 62 children with ADHD were evaluated based on diagnostic subtype. Those with ADHD+SLD were slightly younger, although the difference was not statistically significant (p = 0.085). Disease duration showed a similar, nonsignificant trend (p = 0.128).

Anthropometrically, the ADHD+SLD group were significantly lower in height than the isolated ADHD group (p = 0.026). Weight showed a borderline nonsignificant trend (p = 0.075), while BMI and BMI SDS were similar between groups. Systolic blood pressure was significantly lower in the ADHD+SLD group (p = 0.046), whereas diastolic pressure showed a borderline difference (p = 0.057). No statistically significant differences were observed between the isolated ADHD and ADHD+SLD groups in terms of thyroid hormone levels, including free T_4_, free T_3_, TSH, or overall thyroid function categories (all p > 0.05).

Regarding the categorical variables, the distribution of sexes was similar between the subgroups (p = 0.243). In contrast, the treatment status differed significantly as children with isolated ADHD were more frequently receiving pharmacological treatment than those with ADHD+SLD ( p = 0.009). Mode of delivery (p = 0.275*), incubator admission (p = 0.443*), maternal thyroid medication use during pregnancy (p = 0.417*), additional medication use (p = 0.975*), comorbid medical conditions (p = 0.827*), family history of thyroid disease (p = 0.851*), family history of psychiatric disorders (p = 0.288*), and family history of ADHD/SLD (p = 0.860*) did not differ significantly between groups.

Pubertal stage was also similar between the two ADHD subtypes (p = 0.468*), while thyroid ultrasound findings (p = 0.905*) and thyroid function disorder categories (p = 0.202*) showed no significant group differences.

Table 2 summarizes the continuous and categorical variables comparing the isolated ADHD and ADHD+SLD groups.

### 3.2. Comparison of isolated ADHD, ADHD+SLD, and control groups

A total of 135 children were compared across three diagnostic groups: isolated ADHD, ADHD+SLD, and healthy controls. Age did not differ significantly among the groups (p = 0.428), although the ADHD subgroups were slightly younger than the control group. Birth weight and gestational age were comparable across all three groups.

Sex distribution was comparable between groups (p = 0.752). Treatment status differed substantially (p < 0.001), as would be expected, with all the control participants being untreated, while the treatment rates varied between the isolated ADHD and ADHD+SLD groups.

Mode of delivery did not differ significantly across groups (p = 0.400), while neonatal incubator admission showed a significant group effect (p = 0.019), being more common in the ADHD subgroups than in the control group.

Family history variables—including thyroid disease, psychiatric disorders, and ADHD/SLD—were similar across groups (all p > 0.28). Likewise, prenatal thyroid medication exposure, comorbid medical conditions, and additional medication use showed no significant differences (all p > 0.41). The distribution of pubertal stage was also comparable (p = 0.468).

Thyroid ultrasound findings were similar between the ADHD subgroups (p = 0.905). In contrast, thyroid function categories differed significantly among the three groups (p = 0.019), with abnormal patterns such as subclinical hypothyroidism, isolated T_3_ elevation, and euthyroid Hashimoto’s disease being more frequently observed in the ADHD subgroups compared with the controls.

Significant group differences were observed in height, weight, height SDS, and weight SDS (all p < 0.05), with the control group demonstrating higher anthropometric measurements overall. BMI and BMI SDS, however, were similar across the three groups (p = 0.359 and p = 0.123, respectively).

Cardiovascular findings varied modestly: systolic blood pressure differed significantly between the ADHD subgroups (p = 0.046), whereas diastolic blood pressure (p = 0.057) and resting heart rate (p = 0.633) showed no statistically significant differences.

Among the thyroid parameters, free T_3_ levels differed significantly across the groups in the overall ANOVA (p = 0.002). However, Tukey’s HSD post hoc comparisons did not reveal any statistically significant pairwise differences between the individual diagnostic groups.

Free T_4_ showed borderline significance (p = 0.052), and TSH values exhibited a nonsignificant trend (p = 0.068).

Tukey’s HSD post hoc analyses did not yield statistically significant pairwise differences among the three diagnostic groups (p > 0.05). However, directional trends were observed for higher free T_3_ in ADHD and lower free T_4_ in ADHD+SLD.

When clinically relevant categorical variables were considered, children with isolated ADHD were significantly more likely to be receiving pharmacological treatment than those with ADHD+SLD (p = 0.009). No other categorical clinical variables showed statistically significant differences between the two ADHD subgroups.

To account for possible confounding, age, sex, and treatment status were entered as covariates in all adjusted analyses.

After adjusting for age, sex, and medication status, the ANCOVA indicated a trend-level main effect of diagnostic group on thyroid parameters.

Children with ADHD demonstrated higher free T_3_ levels (F(1, 128) = 4.08, p = 0.046, η^2^ = 0.074) and lower free T_4_ levels (F(1, 128) = 3.14, p = 0.078, η^2^ = 0.040) compared to controls, while TSH was slightly elevated (F(1, 128) = 2.62, p = 0.108, η^2^ = 0.029).

These patterns remained stable after covariate adjustment, suggesting a subtle hypothalamic-pituitary-thyroid (HPT)-axis imbalance in the ADHD group.

The distribution of Free T_3_, Free T_4_, and TSH Levels in the ADHD and Control Groups are shown in Figure.

### 3.3. Medication subgroup analysis

Independent samples t-tests comparing medicated and unmedicated ADHD participants revealed no significant differences in thyroid hormone levels (Free T_3_: p = 0.067; Free T_4_: p = 0.858; TSH: p = 0.479). Unmedicated children showed slightly higher mean Free T_3_ levels, which may reflect a mild normalization of metabolic activity under stimulant treatment (Table 3).

Collectively, these findings point to higher peripheral triiodothyronine (T_3_) and lower thyroxine (T_4_) levels in children with ADHD, independent of medication use, compatible with compensatory HPT-axis activation or altered deiodinase activity.

### 3.4. Correlation analysis

Correlation analyses were performed to examine the relationship between anthropometric indices (BMI and BMI SDS) and thyroid function parameters within the ADHD group. Neither BMI nor BMI SDS showed significant correlations with free T_3_, free T_4_, or TSH levels (all p >0.05). In contrast, a modest positive correlation was observed between free T_3_ and free T_4_ levels (r = 0.289, p = 0.025), consistent with the physiological coupling of thyroid hormones.

## 4. Discussion

In this retrospective comparative study, we examined thyroid function parameters in children with ADHD, ADHD comorbid with SLD, and healthy controls. Our findings reveal a greater frequency of subtle thyroid function abnormalities in children with ADHD when compared with controls, characterized by higher TSH, lower free T_4_, and higher free T_3_ levels, together with increased rates of subclinical hypothyroidism, isolated T_3_ elevation, and euthyroid Hashimoto thyroiditis. Notably, these alterations did not differ between the isolated ADHD and ADHD+SLD subgroups, suggesting that thyroid dysregulation is more strongly associated with the presence of ADHD itself rather than the cooccurrence of learning difficulties. Importantly, BMI and BMI SDS were not correlated with thyroid hormone levels, which suggests that the observed thyroid function differences in ADHD are unlikely to be driven by anthropometric variations.

Although previous studies have reported thyroid hormone differences in various directions, the common theme in the literature—including the present study—is that the HPT axis demonstrates subtle but meaningful alterations in children with ADHD. In a study by Öztürk et al. conducted in Türkiye, higher total T_4_ levels were noted in patients with ADHD and TSH was found to be positively correlated with hyperactivity severity, suggesting an overactive or compensatory HPT response [7]. In contrast, our findings of lower free T_4_ together with higher TSH and elevated free T_3_ indicate a pattern more consistent with relative peripheral hypothyroxinemia accompanied by increased deiodinase activity. These apparent discrepancies in directionality may arise from methodological factors (total versus free hormone assays), differences in age, medication exposure, or variations in thyroid hormone transport and tissue responsiveness. Importantly, both studies align with the broader neuroendocrine model highlighted in recent reviews, which proposes that even minor shifts within the euthyroid range—particularly in the balance of T_3_ and T_4_—may affect the prefrontal and limbic circuits involved in attention and behavioral regulation. Thus, despite differing biochemical profiles, all evidence points toward a shared conclusion: ADHD is associated with subtle HPT-axis dysregulation rather than overt thyroid disease, and this neuroendocrine variability may contribute to the heterogeneity of the cognitive and behavioral symptoms observed in affected children [8]. Consistent with this model, the significantly higher free T_3_ levels observed in both ADHD subgroups in our study may reflect a downstream compensatory response to subtle reductions in free T_4_ or altered central thyroid hormone sensitivity. T_3_ is a biologically active hormone at the neuronal level and increases in peripheral T_3_ may reflect either enhanced deiodinase activity or compensatory mechanisms in the setting of reduced tissue-level thyroid hormone sensitivity [9]. Elevated T_3_ in ADHD has been previously suggested to be a marker of altered HPT-axis regulation, potentially linked to heightened metabolic turnover or stress-related neuroendocrine activation. Previous studies have described a hypermetabolic or hyperarousal phenotype in ADHD, which could theoretically manifest as relative T_3_ dominance. Although causality cannot be inferred from cross-sectional data, our findings support the concept of altered peripheral thyroid hormone dynamics in ADHD [6–8].

We also found that euthyroid Hashimoto thyroiditis was more prevalent in the ADHD group compared with the controls. Autoimmune thyroiditis, even in the absence of overt dysfunction, has been associated with such neuropsychiatric manifestations as impaired attention, mood dysregulation, and slowed processing speed [10]. The proposed mechanisms include the release of inflammatory cytokines, the central effects of autoimmunity, or a subtle disruption of thyroid hormone homeostasis. The presence of Hashimoto thyroiditis in a small subset of ADHD patients in our study suggests that underlying thyroid autoimmunity should be considered during clinical evaluations of children presenting with attentional or behavioral symptoms [10,11].

Beyond these endocrine and immunological findings, our subgroup analysis revealed no significant differences in thyroid parameters between children with isolated ADHD and those with ADHD+SLD. This suggests that comorbid learning difficulties do not further amplify the association between ADHD and subtle HPT-axis variation. Instead, the pattern appears intrinsic to ADHD itself. This aligns with emerging neurodevelopmental literature proposing that ADHD and SLD, while often co-occurring, have partly distinct biological signatures, with ADHD exhibiting stronger ties to arousal regulation, catecholaminergic tone, and metabolic signaling pathways [7,8,12].

It is worth noting that the observed thyroid alterations had only modest effect sizes, indicating that these differences likely represent modulators of the ADHD phenotype rather than primary causes. Slight deviations in thyroid hormone balance may influence attention, processing speed, or behavioral reactivity in susceptible individuals, but do not appear sufficient to produce ADHD independently [13]. This interpretation is supported by the absence of overt thyroid dysfunction in most ADHD cases, as well as prior evidence that subtle endocrine shifts often act synergistically with genetic, neurocognitive, and environmental risk factors rather than functioning as stand-alone etiological drivers [7].

The absence of medication effects on thyroid parameters in our study also offers clinical reassurance. Although stimulant medications can influence appetite, sympathetic activity, and metabolic rate, available evidence suggests that methylphenidate has no consistent or sustained effects on thyroid function in children—when changes are reported, they tend to be small and remain within reference ranges. Some longitudinal studies have described modest decreases in total/free T_4_ during treatment, whereas clinically significant thyroid dysfunction appears rare. In our cohort, thyroid parameters did not differ significantly between the medicated and unmedicated ADHD participants, supporting the interpretation that the observed thyroid patterns are unlikely to be driven solely by stimulant exposure [14,15]. This supports the view that the observed thyroid patterns were more likely linked to underlying physiological traits of ADHD than to treatment-related metabolic changes [7,16]. Nevertheless, medication type and duration could not be fully standardized in this retrospective design and should be considered a potential residual confounder; therefore, future longitudinal studies with larger sample sizes are needed to clarify whether chronic stimulant use has a lasting influence on thyroid regulation.

Our findings also contribute to the broader discussion on mind-body interactions in ADHD. Increasing evidence suggests that metabolic pathways—particularly those involving inflammation, mitochondrial function, and endocrine regulation—may shape cognitive performance and behavioral variability. Thyroid hormones play a critical role in neuronal differentiation, synaptic plasticity, and cortical maturation, particularly in regions implicated in ADHD such as the prefrontal cortex and basal ganglia [17–19]. Subtle reductions in T_4_ availability or relative elevations in T_3_ could therefore influence the neural circuits governing attention, executive control, and arousal. While speculative, this endocrine–neurocognitive interface represents a promising area for future mechanistic research [13].

The clinical implications of our findings should be interpreted with caution. Although current guidelines do not support routine thyroid screening for all children with ADHD, the present findings suggest that thyroid evaluation may be particularly informative in selected cases with atypical clinical features—for example, children with ADHD who also exhibit fatigue, cold intolerance, slowed growth, or a family history of thyroid disease [20]. Additionally, the greater prevalence of euthyroid Hashimoto thyroiditis underscores the potential value of thyroid autoantibody testing in selected patients [21].

Overall, our findings suggest that children with ADHD exhibit subtle but distinct alterations in thyroid hormone dynamics that appear to be independent of comorbid learning difficulties or medication use. Although these changes remain within the euthyroid range, their consistency across our analyses points to a potentially meaningful physiological pattern that may contribute to the neurobiological heterogeneity observed in ADHD. While routine thyroid screening for all children with ADHD is not currently supported, thyroid function evaluation may be considered in selected patients with atypical clinical features, growth concerns, or a family history of thyroid disease. Future longitudinal and mechanistic studies are warranted to clarify the directionality and clinical relevance of these endocrine differences.

### 4.1. Study limitations

This study has several limitations that should be acknowledged. First, the cross-sectional design precludes causal inference. Second, the relatively small size of the ADHD+SLD subgroup may have limited the statistical power to detect subtle between-group differences. Third, missing data related to certain variables may have reduced the overall statistical accuracy.

Additionally, detailed information regarding ADHD symptom severity and response to pharmacological treatment was not consistently available in this retrospective dataset. As a result, we were unable to examine potential associations between symptom severity, treatment response, and thyroid function parameters. Standardized assessments of ADHD severity (e.g., validated rating scales) were not routinely documented, further limiting our analyses.

Moreover, the ADHD subtypes (predominantly inattentive, predominantly hyperactive/impulsive, and combined presentations) could not be reliably differentiated based on available records, and so thyroid function could not be analyzed according to ADHD presentation. Thyroid hormone resistance was not genetically confirmed in this cohort. Cases were classified based solely on biochemical patterns, preventing a definitive diagnosis of resistance to thyroid hormone.

Potential regional iodine deficiency should be considered when interpreting absolute thyroid hormone levels, although the similar environmental exposure across groups minimizes the impact on between-group comparisons.

Finally, differences in age and sex between groups necessitated covariate adjustment, which may have partially attenuated the true biological variability. Future large-scale, longitudinal studies incorporating comprehensive endocrine, genetic, and neurocognitive assessments are warranted to validate and extend our findings.

### 4.2. Conclusion

Children with ADHD demonstrated higher free T_3_, lower free T_4_, and slightly elevated TSH levels, along with increased rates of subclinical thyroid abnormalities compared with the controls. These patterns were not influenced by comorbid SLD or stimulant treatment, indicating that thyroid variation is more closely linked to ADHD itself. While not indicative of overt thyroid disease, subtle alterations in the HPT axis may contribute to the heterogeneous cognitive and behavioral profiles observed in this population.

## Figures and Tables

**Figure:**
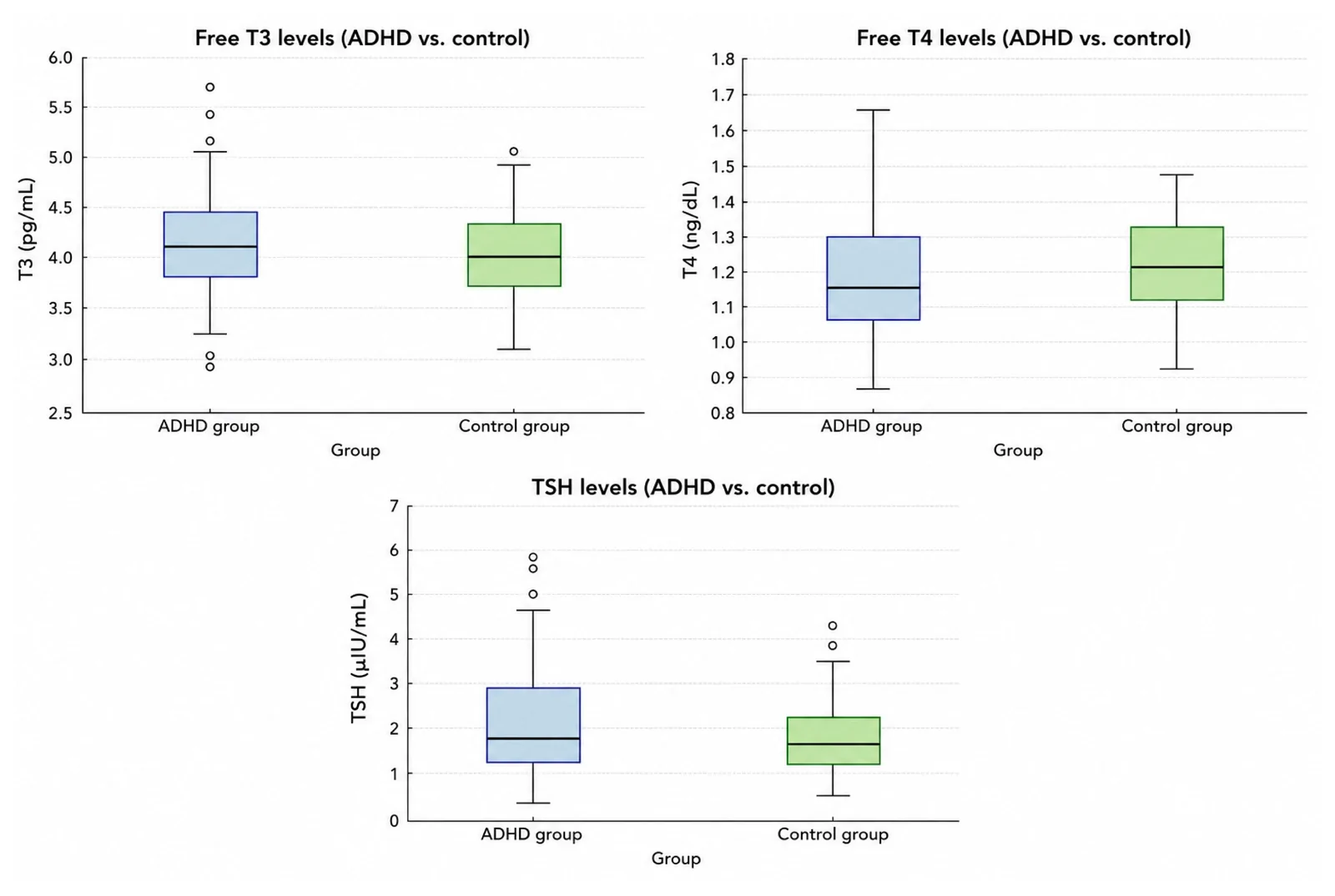
Distribution of free T_3_, free T_4_, and TSH levels in ADHD and control groups.

**Table 1:** Comparison of demographic, anthropometric, and thyroid parameters between ADHD and control groups.

Variable (mean ± SD, or n %)	ADHD (n=62)	Control (n=73)	p-value
**Age (years)**	11.44 ± 2.74	11.97 ± 3.52	0.857*
**Sex**			0.654**↑**
*Female*	36 (58.1%)	48 (65.8%)	
*Male*	26 (41.9%)	25 (34.2%)	
**Mode of delivery**			
*Normal vaginal delivery*	26 (41.9%)	31 (49.2%)	0.414**↑**
*Cesarean section*	36 (58.1%)	32 (50.8%)	
**Birth weight (g)**	3258 ± 614	3273 ± 480	0.879*
**Gestational week**	37.98 ± 5.02	38.38 ± 1.56	0.553*
**Incubator admission**			
*No*	44 (72.1%)	58 (90.6%)	**0.008↑**
*Yes*	17 (27.9%)	6 (9.4%)	
**Height (cm)**	135.85 ± 13.86	149.05 ± 17.69	**<0.001** *
**Height SDS**	−0.25 ± 3.05	0.19 ± 0.99	0.260*
**Weight (kg)**	37.14 ± 14.99	44.84 ± 15.14	**0.003** *
**Weight SDS**	1.83 ± 6.14	0.20 ± 1.12	**0.045** *
**BMI (kg/m** **^2^** **)**	19.12 ± 4.61	19.53 ± 3.72	0.569
**SDS**	0.53 ± 1.24	0.10 ± 1.18	**0.022** *
**Free T** **_4_** ** (ng/dL)**	1.17 ± 0.15	1.22 ± 0.12	**0.020** *
**Free T** **_3_** ** (pg/mL)**	4.20 ± 0.50	3.94 ± 0.45	**0.003** *
**TSH (mIU/L)**	2.21 ± 1.20	1.86 ± 0.78	**0.048** *
**Thyroid function category**			
*Normal*	50 (80.6%)	69 (94.5%)	**0.032↑**
*Subclinical hypothyroidism*	5 (8.1%)	1 (1.4%)	
*Subclinical hyperthyroidism*	1 (1.6%)	0 (0%)	
*Suspected thyroid hormone resistance*	1 (1.6%)	0 (0%)	
*Isolated T_3_ elevation*	2 (3.2%)	3 (4.1%)	
*Euthyroid Hashimoto*	3 (4.8%)	0 (0%)	

* Independent samples t-test **↑**Pearson chi-square test.

Significant p-values are shown in bold.

**Abbreviations:** BMI, body mass index; SDS, standard deviation score; T_3_, triiodothyronine; T_4_, thyroxine; TSH, thyroid-stimulating hormone.

**Table 2:** Comparison of clinical, anthropometric, and thyroid parameters between isolated ADHD and ADHD+SLD groups.

Variable (mean ± SD, or n %)	Isolated ADHD (n=38)	ADHD+SLD (n=24)	p-value
**Age (years)**	11.83 ± 2.50	10.82 ± 1.61	0.085*
**Sex**			0.243**↑**
*Female*	21 (55.3%)	15 (62.5%)	
*Male*	17 (44.7%)	9 (37.5%)	
**Mode of delivery**	0.275**↑**		0.275**↑**
*Normal vaginal delivery*	18 (47.4%)	8 (33.3%)	
*Cesarean section*	20 (52.6%)	16 (66.7%)	
**Birth weight (g)**	3373 ± 497	3066 ± 744	0.088*
**Gestational week**	37.89 ± 6.32	38.13 ± 1.62	0.862*
**Incubator admission**	0.443**↑**		0.443**↑**
*No*	28 (75.7%)	16 (66.7%)	
*Yes*	9 (24.3%)	8 (33.3%)	
**Height (cm)**	139.00 ± 14.98	130.99 ± 10.48	**0.026** *
**Height SDS**	0.27 ± 1.05	−1.03 ± 4.64	0.105*
**Weight (kg)**	39.90 ± 16.01	32.90 ± 12.41	0.075*
**Weight SDS**	1.10 ± 2.97	2.94 ± 9.07	0.256*
**BMI (kg/m** **^2^** **)**	19.68 ± 4.70	18.25 ± 4.42	0.239*
**BMI SDS**	0.57 ± 1.23	0.46 ± 1.28	0.723*
**Free T** **_4_** ** (ng/dL)**	1.16 ± 0.16	1.18 ± 0.15	0.547*
**Free T** **_3_** ** (pg/mL)**	4.12 ± 0.46	4.33 ± 0.53	0.116*
**TSH (mIU/L)**	2.33 ± 1.28	2.01 ± 1.06	0.317*
**Thyroid function category**			0.202**↑**
*Normal*	32 (84.2%)	18 (75.0%)	
*Subclinical hypothyroidism*	4 (10.5%)	1 (4.2%)	
*Subclinical hyperthyroidism*	1 (2.6%)	0 (0%)	
*Suspected thyroid hormone resistance*	0 (0%)	1 (4.2%)	
*Isolated T_3_ elevation*	0 (0%)	2 (8.3%)	
*Euthyroid Hashimoto*	1 (2.6%)	2 (8.3%)	

* Independent samples t-test **↑**Pearson chi-square test.

Significant p-values are shown in bold.

**Abbreviations:** BMI, body mass index; SDS, standard deviation score; T_3_, triiodothyronine; T_4_, thyroxine; TSH, thyroid-stimulating hormone.

**Table 3:** Adjusted group comparisons and effect sizes of thyroid function parameters.

Parameter	ADHD	Control	ANCOVA F(1, 128)	p-value	η^2^	Cohen’s *d*	Interpretation
**Free T** **_3_** ** (pg/mL)**	4.20 ± 0.50	3.94 ± 0.45	4.08	0.046	0.074	0.56	Higher in ADHD
**Free T** **_4_** ** (ng/dL)**	1.17 ± 0.15	1.22 ± 0.12	3.14	0.078	0.040	−0.40	Lower in ADHD
**TSH (mIU/L)**	2.21 ± 1.20	1.86 ± 0.78	2.62	0.108	0.029	0.34	Slightly higher in ADHD

All values adjusted for age, sex, and medication status. Cohen’s d effect sizes: 0.2 = small, 0.5 = medium, 0.8 = large.

## Data Availability

Since this is a retrospective study, the data supporting the findings of this study are not publicly available due to ethical and legal restrictions related to patient confidentiality. The data can be made available by the corresponding author upon reasonable request and with the permission of the relevant institutional authorities.

## Footnotes

1 Alahmari FS (2025). ADHD in school-age children: Overview [online]. Website https://www.intechopen.com/chapters/1221550 [accessed 12 July 2025].
